# Bioassay-Guided Isolation of Antiproliferative Compounds from *Limbarda crithmoides* (L.) Dumort

**DOI:** 10.3390/molecules25081893

**Published:** 2020-04-20

**Authors:** Sabrina Adorisio, Laura Giamperi, Anahi Elena Ada Bucchini, Domenico Vittorio Delfino, Maria Carla Marcotullio

**Affiliations:** 1 Department of Medicine, Foligno Nursing School, University of Perugia, 06034 Foligno, Italy; adorisiosabrina@libero.it; 2 Department of Biomolecular Sciences, Section of Biochemistry and Biotechnology, University of Urbino, 61029 Urbino, Italy; laura.giamperi@uniurb.it (L.G.); elena.bucchinianahi@uniurb.it (A.E.A.B.); 3 Department of Medicine, Section of Pharmacology, University of Perugia, 06132 Perugia, Italy; 4 Department of Pharmaceutical Sciences, Università degli Studi di Perugia, 06123 Perugia, Italy

**Keywords:** *Inula crithmoides*, *Limbarda crithmoides*, Asteraceae, antiproliferative activity, OCI-AML3

## Abstract

*Limbarda crithmoides* (L.) Dumort (Asteraceae) *n*-hexane extract displayed high cell proliferation inhibitory activity against acute myeloid leukaemia cells (OCI-AML3) and was therefore subjected to a bioassay-guided multistep separation procedure. Two thymol derivatives, namely 10-acetoxy-8,9-epoxythymol tiglate (**1**) and 10-acetoxy-9-chloro-8,9-dehydrothymol (**2**), were isolated and identified by means of NMR spectroscopy. Both of them exhibited a significant dose-dependent inhibition of cell proliferation.

## 1. Introduction

The genus *Limbarda* (Asteraceae), formerly included in the genus *Inula*, comprises two accepted species: *L. crithmoides* (L.) Dumort., and *L. salsoloides* (Turcz.) Ikonn [1]. *L. crithmoides* is a halophyte plant, commonly known as *Inula crithmoides* (L.), that is widespread across the salt marshes and sea cliffs of the Mediterranean Sea, French Atlantic coasts, English channel and western European seaboards [1]. *L. crithmoides* is a common edible plant. Its leaves, eaten raw or boiled, are a good source of protein, amino acids, fibers, vitamins and other components, and traditionally formed an important part of the Lebanese diet [2]. Young shoots of *L. crithmoides* are also consumed as pickles in vinegar in Spain [3], while its raw tops are added to salads in the Basilicata region of southern Italy [4]. *I. crithmoides* biological activities have been reviewed, along with other eight Asteraceae representative species [5]. Essential oil composition has been widely explored [6,7,8], and it showed to be dependent on the region where plants grow [8].

Extracts of *L. crithmoides* have been applied to crops and weeds to investigate their properties, which confirmed their herbicidal potency [9]. It was also reported that the extracts of callus cultures show good antifungal activity against *Alternaria solani* and *Phytophthora cryptogea* [10]. In a previous work, methanol and hexane extracts of the aerial parts of this plant were found to reduce the radial growth of *A. solani* and *P. cryptogea*, and they also show weak antifungal activity against fungi of the *Fusarium* genus [11]. The antioxidant activity of *L. crithmoides* has been widely investigated [11,12,13,14], and seems to be directly correlated with the presence of phenolic metabolites such as quinic acid derivatives [12,15,16,17]. For example, the ethyl acetate fraction of a methanolic extract of *I. crithmoides* showed in vitro and in vivo hepatoprotective activity against carbon tetrachloride (CCl_4_)-induced liver injury through antiradical and antioxidant activities [18]. In addition to antifungal activity, antimicrobial as well as antileishmanial activities of *L. crithmoides* extracts have also been reported [15,19,20,21].

As part of our ongoing research into antiproliferative compounds derived from natural sources [22,23,24,25,26], a phytochemical investigation of *L. crithmoides* was undertaken to isolate active compounds by means of a bioassay-guided fractionation of active extracts against the Ontario Cancer Institute-Acute Myeloid Leukemia-3 (OCI-AML3) acute myeloid leukaemia cell line.

## 2. Results and Discussion

*L. crithmoides* (*I. crithmoides*) is a halophyte plant present in areas of high salinity [27,28] that represents an important agricultural crop [29,30] for its use as a food and for biological activities. Proximate composition of different extracts [6,7,31] showed the presence of saccharides [32] and proteins [33]. The most representative metabolites of this plant are phenolics [16,17,20,34,35,36,37] that are responsible of the good antioxidant and antiradical activity of extracts [10,11,12,13,15,38]. *L. crithmoides* essential oil and extracts were studied for their antimicrobial and antifungal activities [10,15,19,20,33,38]. *Inula* spp. have been investigated in depth for their cytotoxic/antiproliferative activity [39,40,41]. Despite the large number of biological studies undertaken on *L. crithmoides* [16,18,34,42], nothing has been reported so far about the cytotoxic/antiproliferative activity of this species and this prompted us to investigate the activity of *L. crithmoides* extracts against the OCI-AML3 cell line.

In a preliminary study, 24 h of treatment with 100 and 200 µg/mL of the methanolic extract (M) of *L. crithmoides* induced a significant decrease in OCI-AML3 cell number (Figure S1A, left panel, Supplementary Material). The augmentation observed with 200 µg/mL was accompanied by a significant increase in apoptosis (Figure S1A, right panel and S1B, Supplementary Material), associated with an accumulation of cells in the G0/G1 phase and a consequent decrease of cells in S and G2/M phases of the cell cycle (Figure S2A,B, Supplementary Material). The active M extract was subjected to solvent–solvent partitioning, yielding *n*-hexane (H), methylene chloride (DCM) and aqueous MeOH-soluble extracts. Both H and DCM extracts were able to significantly decrease the OCI-AML3 cell number compared to the vehicle (control) at concentrations of 15 or 10 μg/mL (with both extracts, Figure S3A, left panel, Supplementary Material). Analysis of apoptotic cell death showed a significant increase in apoptosis with 10 and 15 µg/mL of the H extract when compared with DCM extract (Figure S3A, right panel, and S3B, Supplementary Material). An analysis of the cell cycle revealed a significant increase in cells in the G0/G1 phase (Figure S4A, left panel, Supplementary Material) and fewer cells in S and G2/M phases of the cell cycle (Figure S4A, middle and right panels, respectively, Supplementary Material) with concentrations of 10 and 15 µg/mL for both fractions. Figure S4B shows a representative experiment of the experiment series quantified in Figure S4A. Thus, the H and DCM extracts were at least 10-fold more active than the M extract (Figures S3 and S4, Supplementary Material), and the H extract was more potent than the DCM extract in increasing apoptotic cell death. For this reason, we decided to continue only with the H extract.

**Figure 1:**
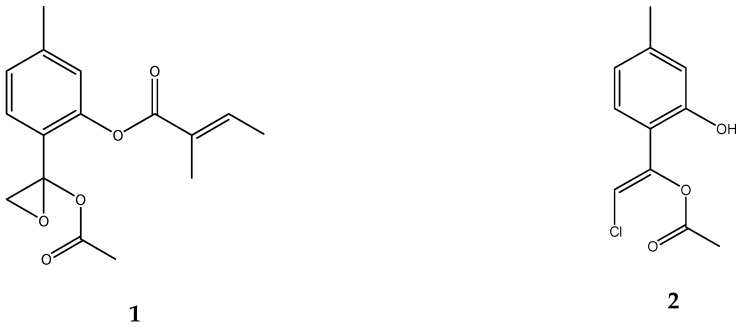
Chemical structures of active compounds **1** and **2**.

The serial bioassay-guided chromatographic procedures led to the isolation of two active compounds (**1** and **2**; Figure 1). Using NMR spectroscopic data, compound **1** was identified as 10-acetoxy-8,9-epoxythymol tiglate, previously isolated from *Athrixia* spp. [43], *Schkuhria multiflora* Hook & Arn. [44] and *Eupatorium cannabinum* L. [45], and compound **2** was characterized as 10-acetoxy-9*Z*-chloro-8,9-dehydrothymol, previously isolated from *Arnica sachalinensis* (Regel) A. Gray [46] (Figures S5–S8, Supplementary Material). The Z configuration of the double bond was confirmed by NOESY experiment (Figure S9). Thymol derivatives are quite common in different *Inula* spp. [47,48,49], and it is known that *L. crithmoides* produces chlorinated thymol derivatives [37]. However, as far as we know, this is the first time that compounds **1** and **2** have been isolated from *L. crithmoides* (*I. crithmoides*). The two pure compounds were tested for their biological activity.

**Figure 2:**
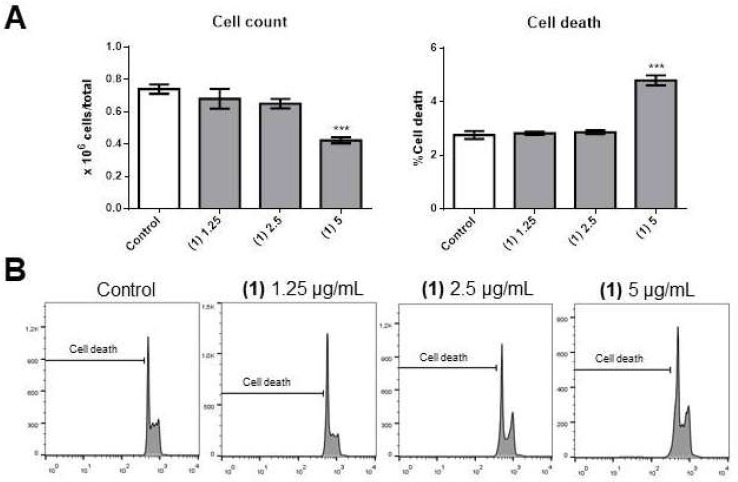
Effects of compound **1** on OCI-AML3 cell number and apoptotic cell death. (**A**) Bars represent the cell number (left panel) or the percentage of apoptotic cells after 24 h of treatment with control vehicle (Control) or 1,25 [(1) 1.25)], 2,5 [(1) 2.5] or 5 [(1) 5] µg/mL of compound **1**. (**B**) Flow cytometry analyses of a representative experiment. Data from three independent experiments are reported as mean ± SEM. *** *p* < 0.001.

**Figure 3:**
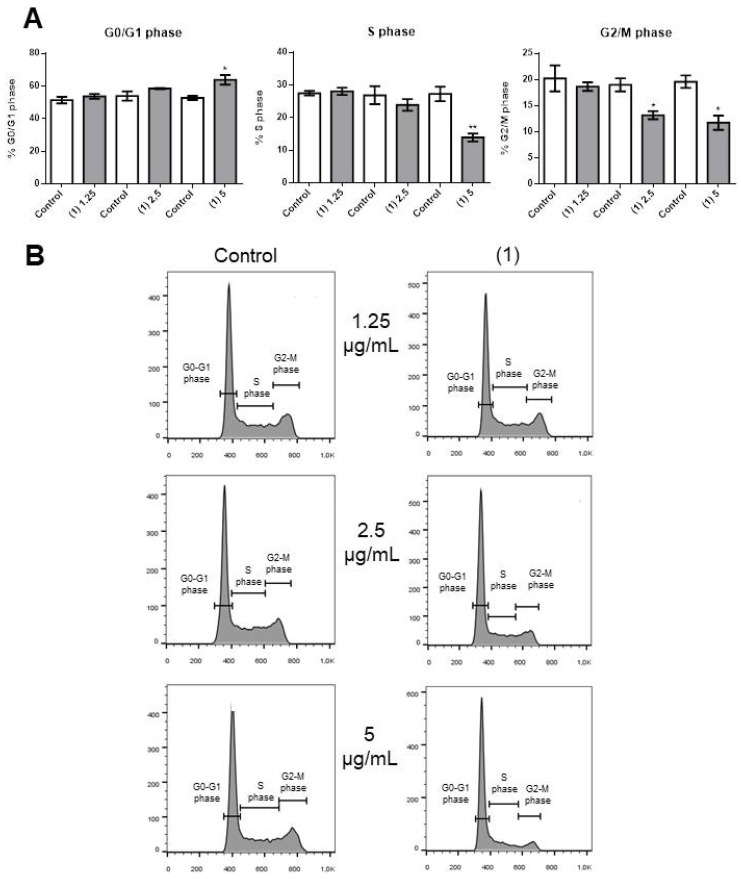
Effects of compound **1** on OCI-AML3 cell cycle progression. (**A**) Bars represent the percentage of cells in G0/G1 (left panel), S (middle panel), or G2/M (right panel) phases after 24 h of treatment with control vehicle (Control) or 1.25 [(1) 1.25], 2.5 [(1) 2.5] or 5 [(1) 5] µg/mL of compound **1**. (**B**) Flow cytometry analyses of a representative experiment. Data from three independent experiments are reported as mean ± SEM. * *p* < 0.05, ** *p* < 0.01.

Compound **1** caused a significant decrease in OCI-AML3 cell number at 5 µg/mL (Figure 2A, left panel). This was possibly the consequence of a significant increase in apoptosis (Figure 2A, right panel, and Figure 2B) and blocking of cell cycle progression, as we observed significant accumulation of cells in the G0/G1 phase (Figure 3A, left panel) and fewer cells in the S (middle panel) and G2/M (right panel) phases of the cell cycle.

**Figure 4:**
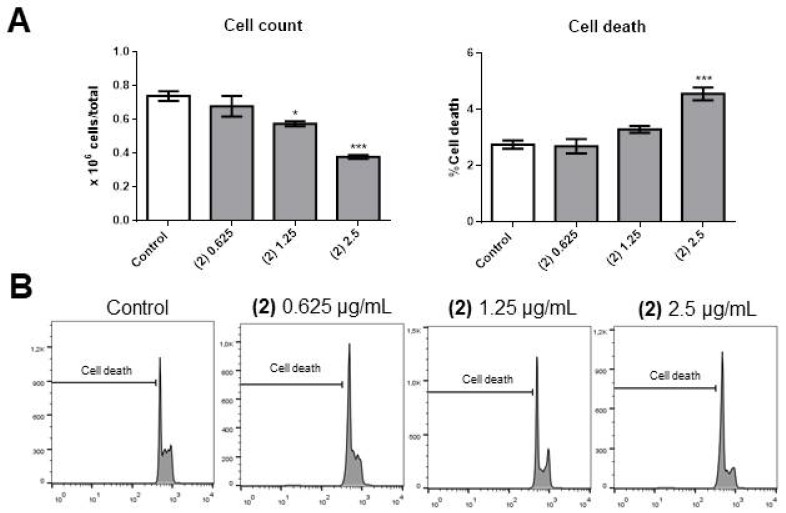
Effects of compound **2** on OCI-AML3 cell number and apoptotic cell death. (**A**) Bars represent the cell number (left panel) or the percentage of apoptotic cells after 24 h of treatment with control vehicle (Control) or 0.625 [(2) 0.625)], 1.25 [(2) 1.25] or 2.5 [(2) 2.5] µg/mL of compound **2**. (**B**) Flow cytometry analyses of a representative experiment. Data from three independent experiments are reported as mean ± SEM. * *p* < 0.05; *** *p* < 0.001.

**Figure 5:**
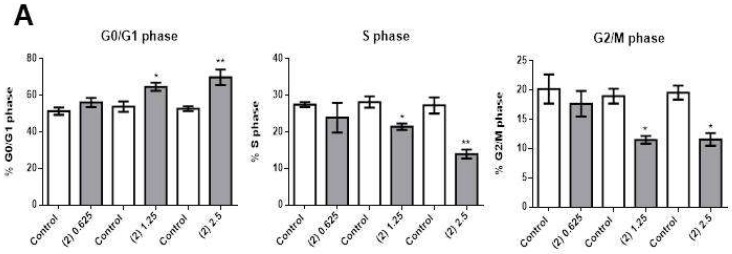

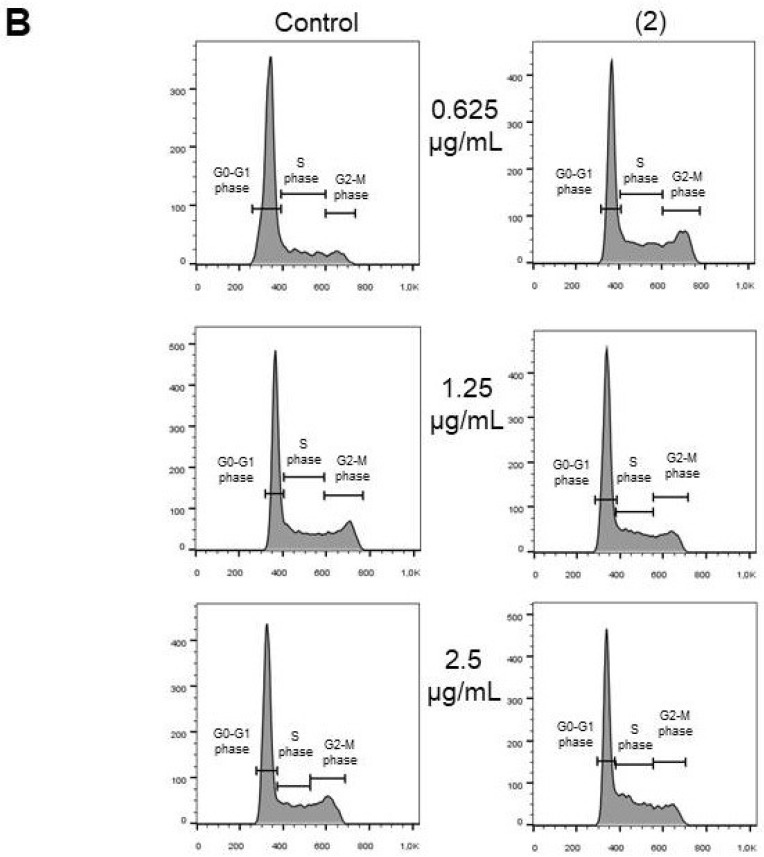
Effects of compound **2** on OCI-AML3 cell cycle progression. (**A**) Bars represent the percentage of cells in G0/G1 (left panel), S (middle panel), or G2/M (right panel) phases after 24 h of treatment with control vehicle (Control) or 0.625 [(2) 0.625], 1.25 [(2) 2.25] or 2.5 [(2) 2.5] µg/mL of compound **2**. (**B**) Flow cytometry analyses of a representative experiment. Data from three independent experiments are reported as mean ± SEM. * *p* < 0.05, ** *p* < 0.01.

Compound **2** caused a significant decrease in cell number at concentrations as low as 1.25 µg/mL (Figure 4A, left panel), and there was a significant increase in apoptosis in cells treated with 2.5 µg/mL (Figure 4A, right panel, and Figure 4B). Compound **2** blocked the cell cycle progression at G0/G1 (Figure 5A, left panel), with 1.25 µg/mL causing a consequent significant decrease in cells in the S (Figure 5A, middle panel, and Figure 5B) and G2/M (Figure 5A, right panel, and Figure 5B) phases of the cell cycle.

**Figure 6:**
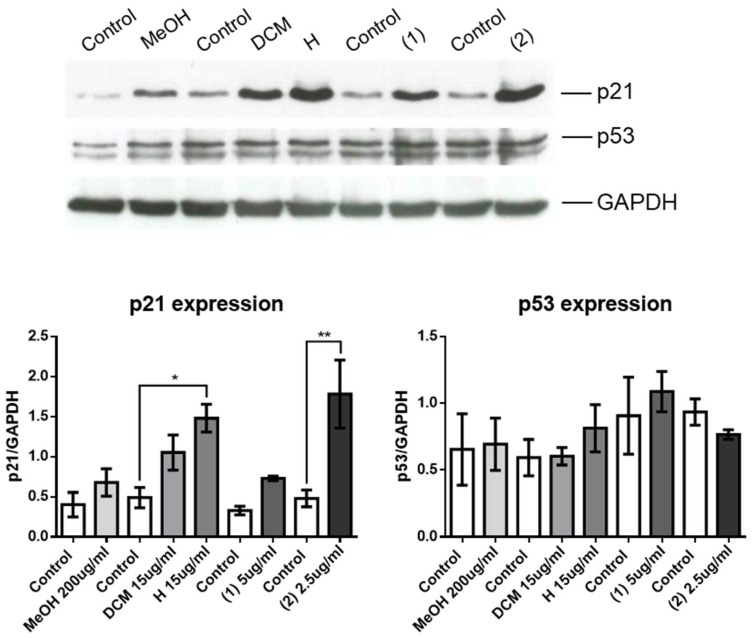
Effects of extracts and compounds on expression of proteins involved in the cell cycle. Upper panel, western blot analysis illustrating expression of p21, p53 and GAPDH using cell lysates extracted from OCI-AML3 cells treated with vehicle (Control), M (MeOH), DCM (DCM), H (H) extracts and compound **1** (1) or **2** (2) for 24 h. Western blots are representative of three independent experiments. Lower panels, Quantification of experiments shown in upper panel with the indication of the concentration used. Data from three independent experiments are reported as mean ± SEM. * *p* < 0.05, ** *p* < 0.01.

Thus, compounds **1** and **2** were very effective in decreasing the number of OCI-AML3 cells through both increasing apoptosis and blocking cell proliferation. Compound **2** was more potent than compound **1** as it exerted inhibitory effects at a concentration as low as 1.25 µg/mL. The increased expression of p21 is largely associated with the arrest of cell cycle by two different pathways. The first is a p53-dependent and the second is p-53 -independent pathway. This prompted us to examine the expression of both p21 and p53 in OCI-AML3 cells untreated or treated with the M, DCM, H extracts and the compounds **1** and **2** isolated from the latter as a possible mechanism of cell cycle arrest since these pathways regulates mitotic progression and promotes cellular stress response [50]. We used Western blotting to measure expression of p21 in control and treated cells. As shown in Figure 6, upper panel, all the tested extracts and compounds induced an upregulation of p21, but only with the H extract and with compound **2** the increase of p21 was significant compared to controls (Figure 6, lower left panel). Because p21 is regulated through either p53-dependent or -independent pathways, we also measured expression of p53, which did not change after treatment with any of the extracts or compounds (Figure 6, upper and lower right panels).

These results suggest that the H extract induced a significant activation of a p21-dependent, p53-independent pathway in OCI-AML3 cells and that compound **2** was responsible of this activity and the associated cell cycle arrest. Notably, the p21-dependent, p-53-independent pathway has been shown to be associated with an increased apoptosis, as is evident in our system, in contrast to the p21-dependent, p53-dependent pathway that promotes the translocation of p21 from the nucleus to the cytoplasm thus determining an anti-apoptotic effect absent in our study [22].

It is known from previous studies that compounds isolated from *Inula* species may have antiproliferative effect on a different leukaemia cells [51,52,53,54,55,56,57,58,59] but, as far as we know, only one work describing the activity of *Inula* extract on an acute myeloid leukaemia (KG1a cell line) has been reported [60]. This work highlighted the effect of *Inula* compounds on induction of apoptosis by the mitochondria-dependent pathway. Most of the *Inula* metabolites tested were sesquiterpenolides. On the other hand, thymol and thymol derivatives as well as extracts and essential oils containing these compounds [61,62,63,64] are known to be cytotoxic.

## 3. Materials and Methods

### 3.1. General Chemical Material

NMR spectra were recorded using Avance DRX-400 and DPX-200 spectrometers (Bruker, Milan, Italy) operating at frequencies of 400 MHz (^1^H) and 100 MHz (^13^C) and 200 MHz (^1^H) and 50 MHz (^13^C), respectively The spectra were measured in CDCl_3_. The ^1^H- and ^13^C-NMR chemical shifts (δ) are expressed in ppm with reference to the solvent signals (CDCl_3_, δ_H_ 7.26 and δ_C_ 77.1). Coupling constants are given in Hz. NOESY (2D- NOE) experiments were executed on the Bruker Avance DRX-400 instrument. Preparative TLC was performed using pre-coated silica gel 60 F-254 plates (10 × 20 cm, Merck, Sigma-Aldrich, Milan, Italy) using *n*-hexane-acetone 8.5:1.5 as the eluent. Spots were visualized under UV light. Compounds were recovered from the stationary phase by washing five times with CH_2_Cl_2_ (DCM). Column chromatography was performed using MN Kiesegel 60 (70–230 mesh, Macherey-Nagel, Fisher Scientific, Milan, Italy). Fractions were monitored by TLC (Silica gel 60 F254; Merck), and spots on TLC were visualised under UV light and after staining with *p*-anisaldehyde-H_2_SO_4_-EtOH (1:1:98) followed by heating at 110 °C. All solvents used were of analytical grade and were purchased from VWR (VWR, Milan, Italy). Anhydrous Na_2_SO_4_ was purchased from Scharlau S.L. (Milan, Italy).

### 3.2. Plant Material

Aerial parts of *L. crithmoides* were collected during the flowering period (August 2016) in Fano, Urbino, Italy. Specimen collection was restricted to coastal habitats, nearly always within the reach of sea spray. Specimens were authenticated by Dr Laura Giamperi (University of Urbino). Voucher specimens have been deposited at the Herbarium of the Botanic Garden of the University of Urbino (GS 203).

### 3.3. Extraction and Isolation Procedure

Air-dried and finely powdered plant material (50 g) was extracted by maceration in methanol (MeOH) (3 × 500 mL for 24 h each). The combined extracts gave 12.87 g of active MeOH extract (M; yield 26%). The extract was dissolved in 10 mL of MeOH, diluted with H_2_O (100 mL), then subjected to solvent-solvent partition between *n*-hexane (3 × 50 mL; H extract; 6.2% yield) and methylene chloride (CH_2_Cl_2_) (3 × 50 mL; DCM extract; 3.4% yield). The H extract, which was the most active, was subjected to silica gel column chromatography (2 × 40 cm) under conditions of gradient elution using a mixture of CH_2_Cl_2_ in *n*-hexane (0→100%). Using *n*-hexane (1 L), we obtained four fractions: fraction 1 (IC-H-F1; 13 mg), using *n*-hexane-CH_2_Cl_2_ (1:1) (1.2 L) we obtained F2 (IC-H-F2, 280 mg), using CH_2_Cl_2_ (1.25 L) we obtained F3 (IC-H-F3, 120 mg) and F4 (IC-H-F4, 190 mg). Finally, the column was washed with 500 mL of MeOH (200 mg). The obtained fractions were tested for their antiproliferative activity using the same procedure for the extracts. An aliquot of the most active IC-H-F3 (109 mg) was further purified by silica gel column (1 × 20 cm) chromatography using CH_2_Cl_2_ as a solvent. Fractions (5 mL) were collected, evaluated by TLC and combined as a result of their similar appearance, yielding four pooled fractions: IC-H-F3(1), 36 mg; IC-H-F3(2), 52 mg; IC-H-F3(3), 6 mg; IC-H-F3(4), 6 mg. A solution of the IC-H-F3(1) fraction in 1 mL of DCM was purified by semipreparative TLC using *n*-hexane-acetone 8.5:1.5 as the eluent, resulting in the isolation of compound **1** (8 mg) and compound **2** (12 mg) (Figure S9, Supplementary Material). Each compound was identified by direct comparison of spectral properties, MS, ^1^H-NMR and ^13^C-NMR, with those of the authentic compounds in the literature [19,22].

### 3.4. Cell Line Culture and Characterisation

The antiproliferative activity of the two compounds under consideration was tested on the acute myeloid leukaemia (AML) cell line OCI-AML3 [65]. OCI-AML3 cells were maintained in Roswell Park Memorial Institute (RPMI) 1640 medium with 10% foetal bovine serum (FBS), 100 U/mL penicillin, and 100 μg/mL streptomycin at 37 °C and 5% CO_2_. Cells were purchased from ATCC (LGC Standards S.r.l., Sesto San Giovanni, Milan, Italy), kept at logarithmic growth and cultured in 24-well plates to assess their number and morphology. Cultures kept at 2 × 10^5^ cells/mL were treated with different concentrations of DMSO or the test compounds at the final concentrations reported in the figures. These reported concentrations were chosen based on preliminary experiments. After 24 h, the cell number was quantified using a haemocytometer.

### 3.5. Analysis of Cell Viability and Cell Cycle Progression

Cell viability and cell cycle progression were examined by flow cytometry to measure the amount of DNA in nuclei stained with propidium iodide (PI; Sigma-Aldrich, Milan, Italy), with the exclusion of necrotic cells by forward light scatter (FSC) [66]. Briefly, cells were harvested by centrifugation and gently resuspended in 1.5 mL hypotonic PI solution (50 µg/mL in 0.1% sodium citrate plus 0.1% Triton X-100). Tubes were kept in the dark at 4 °C for 30 min. PI fluorescence of individual nuclei was measured by flow cytometry using a Coulter Epics XL-MCLTM flow cytometer (Beckman Coulter, Cassina De’ Pecchi, Milan, Italy) and analysed using FlowJo_V10 software (BD Biosciences, Milan, Italy).

## 4. Conclusions

This study showed that the M extract of *L. crithmoides* has cell proliferation inhibitory activity against acute myeloid leukaemia cells (OCI-AML3) and solvent partition showed that the H fraction was more active than the DCM fraction. For this reason, we decided to continue testing the H fraction. Chromatographic purification of fraction H led to the isolation of two active thymol derivatives, **1** and **2**. Compound **2** was shown to be highly active. As far as we know, this is the first report of the antiproliferative activity of chlorinated thymol derivatives. The DCM fraction, to a minor extent, was shown to still be active, and for this reason we think it would be interesting to explore its composition and the antiproliferative activity of its fractions and compounds.

## Supplementary Materials

NMR spectra of the active compounds and biological activity assay result informations are available online.
