# Supplementary material for: Bioassay-Guided Isolation of Antiproliferative Compounds from Limbarda crithmoides (L.) Dumort
Source: Molecules. 2020 Apr 20;25(8):1893. doi: 10.3390/molecules25081893 (PMC7221903; doi:10.3390/molecules25081893)
Supplement: Supplementary file 1 [file molecules-25-01893-s001.pdf]

Supporting Information

Sabrina Adorisio [1], Laura Giamperi [2], Anahi Elena Ada Bucchini [2], Domenico Vittorio Delfino [1,3,*] and Maria Carla Marcotullio [4,*]

1 Foligno Nursing School, Department of Medicine, University of Perugia, 06034 Foligno, Italy; adorisiosabrina@libero.it

2 Department of Biomolecular Sciences, Section of Biochemistry and Biotechnology, University of Urbino , 61029 Urbino, Italy; laura.giamperi@uniurb.it (L.G.), elena.bucchinianahi@uniurb.it (A.E.A.B.)

3 Department of Medicine, Section of Pharmacology, University of Perugia, 06132 Perugia, Italy

4 Department of Pharmaceutical Sciences, Università degli Studi di Perugia, 06123 Perugia, Italy

* Correspondence: domenico.delfino@unipg.it (D.V.D.), mariacarla.marcotullio@unipg.it (M.C.M.); Tel.: +39-075-585-8186 (D.V.D.), Tel.: +39-075-585-5100 (M.C.M.)

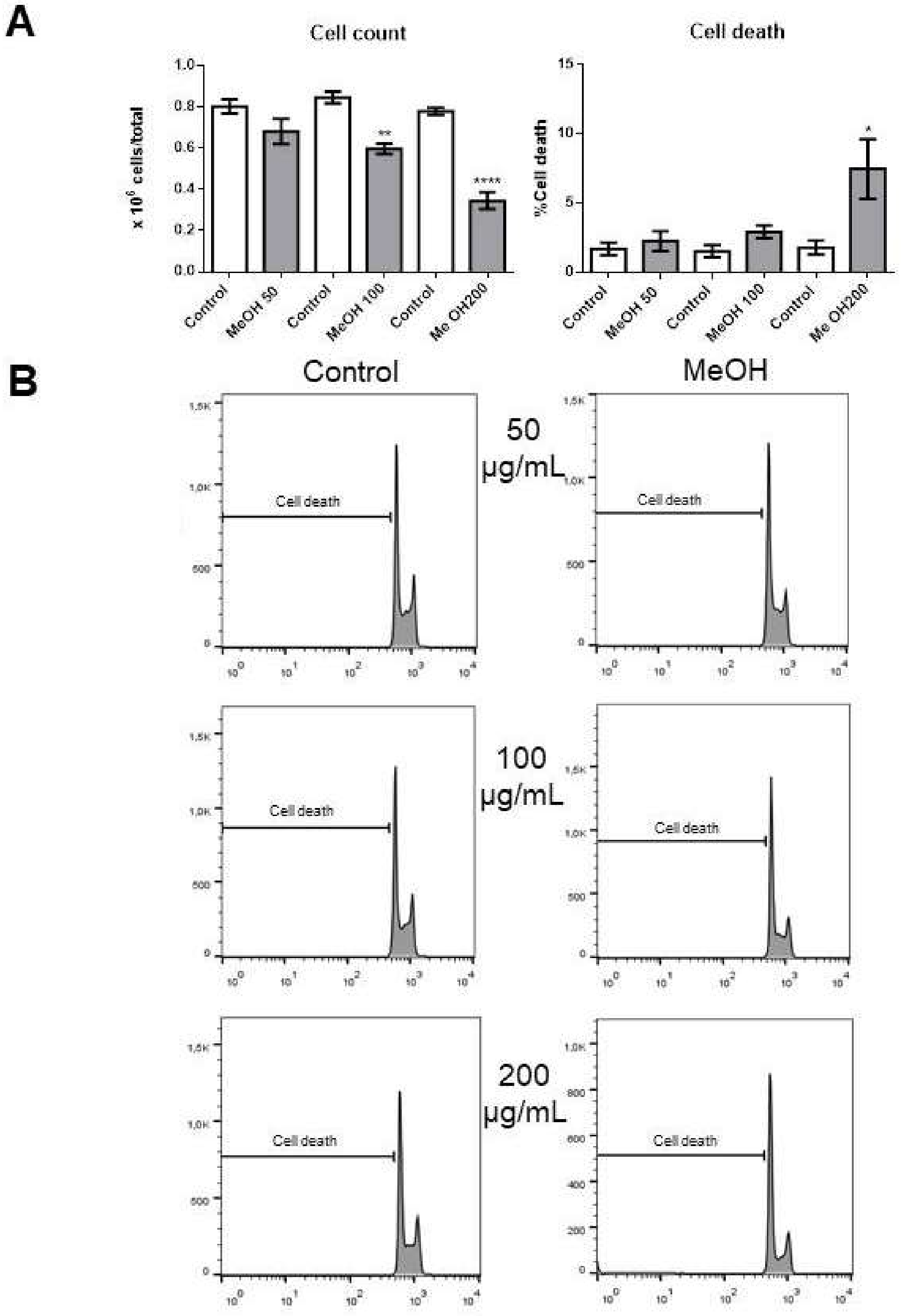

Figure S1. Effects of methanol extract (M) of *L. crithmoides* on OCI-AML3 cell number and apoptotic cell death. **A.** Bars represent the cell number (left panel) or the percentage of apoptotic cells after 24 h of treatment with control vehicle (Control) or 50 (MeOH 50), 100 (MeOH 100) or 200 (MeOH 200) µg/mL of the methanol extract (M). **B**. Flow cytometry analyses of a representative experiment. Data from three independent experiments are reported as mean ± SEM. * < 0.05; ** < 0,01; *** < 0,001.

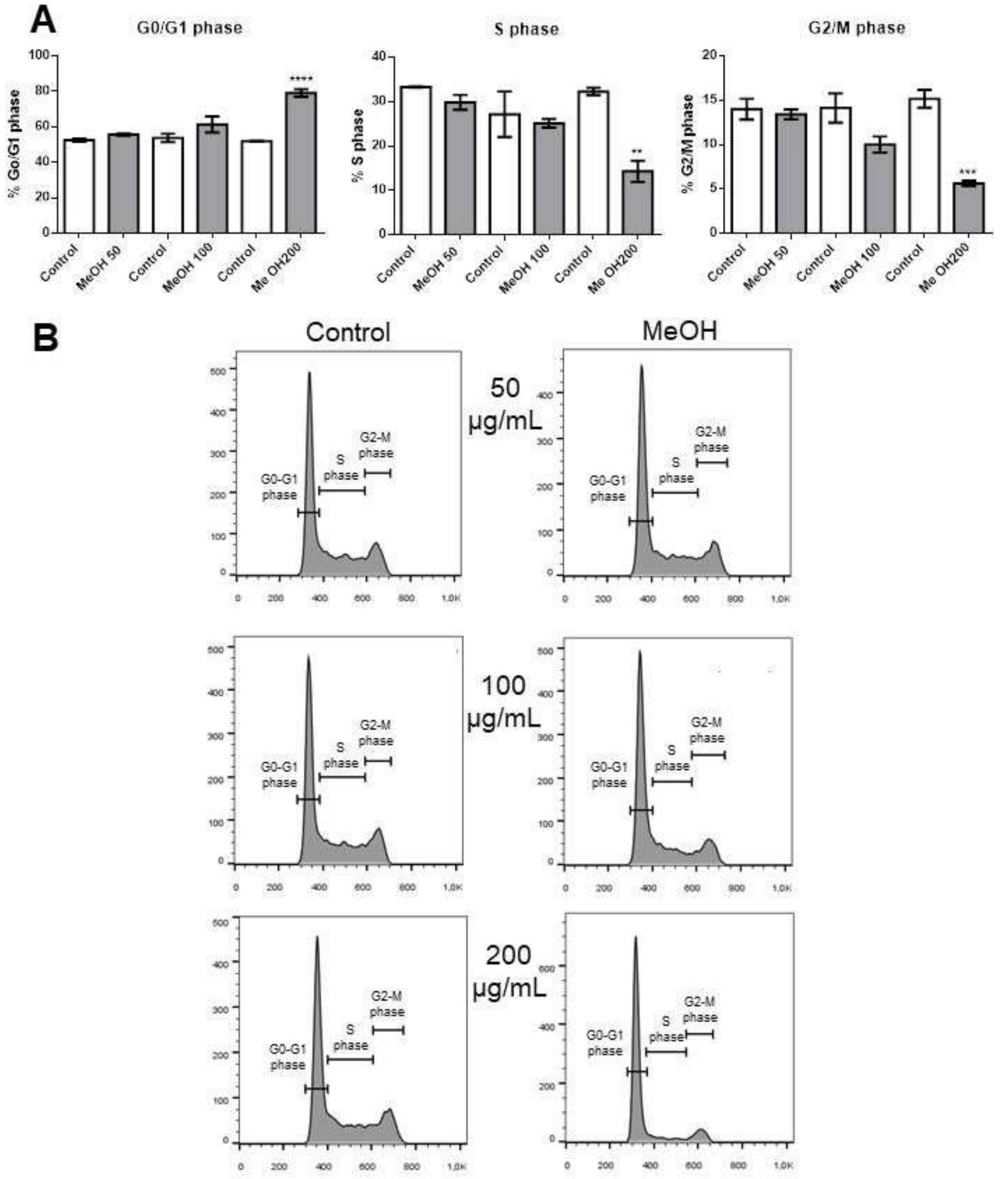

Figure S2. Effects of methanol extract (M) of *L. crithmoides* on OCI-AML3 cell cycle progression. **A.** Bars represent the percentage of cells in G0/G1 (left panel), S (middle panel), or G2/M (right panel) phases after 24 h of treatment with control vehicle (Control) or 50 (MeOH 50), 100 (MeOH 100) or 200 (MeOH 200) µg/mL of the methanol extract (M). **B**. Flow cytometry analyses of a representative experiment. Data from three independent experiments are reported as mean ± SEM. ** < 0,01; *** < 0,001.

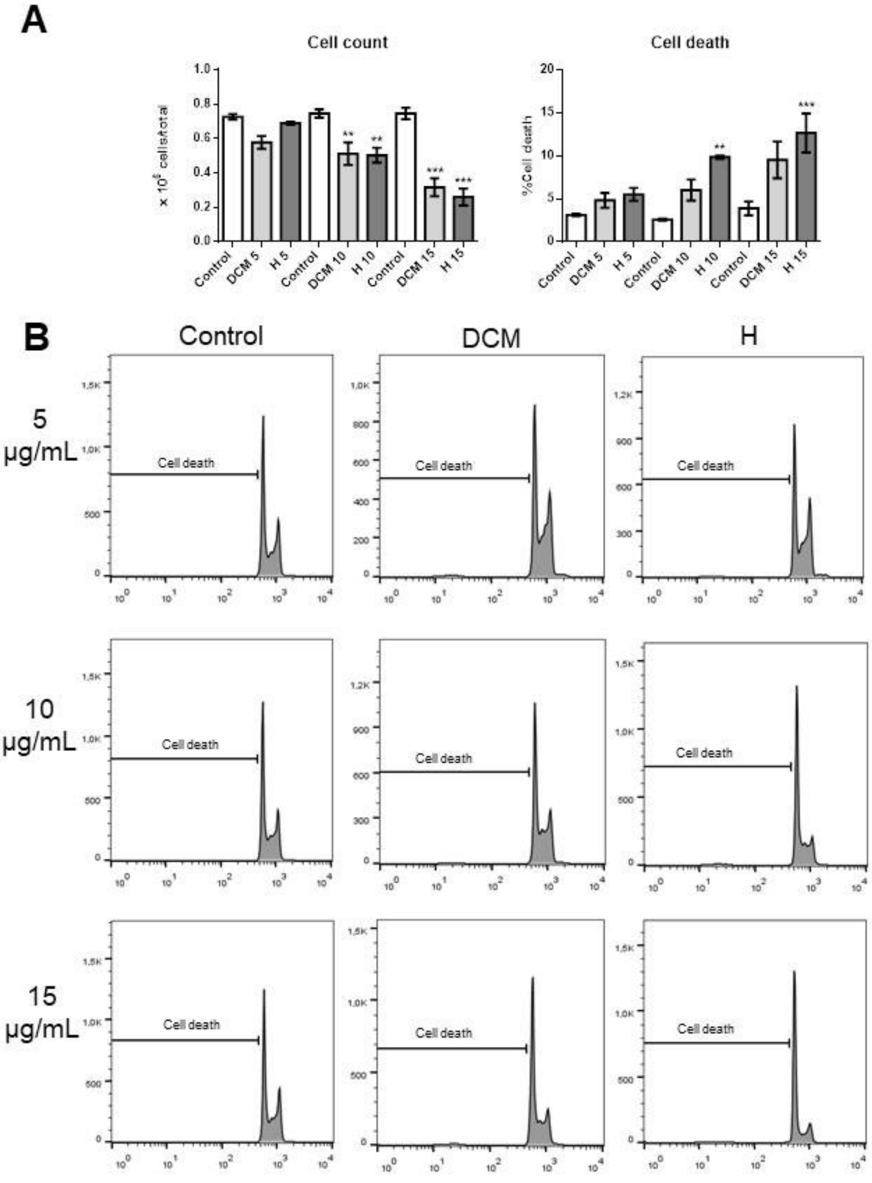

Figure S3. Effects of DCM or H extracts of *L. crithmoides* on OCI-AML3 cell number and apoptotic cell death**. A.** Bars represent the cell number (left panel) or the percentage of apoptotic cells after 24 h of treatment with control vehicle (Control) or 5 (DCM 5; H 5), 10 (DCM 10; H 10) or 15 (DCM 15; H 15) µg/mL of the DCM or H extracts, respectively. **B**. Flow cytometry analyses of a representative experiment. Data from three independent experiments are reported as mean ± SEM. * < 0.05; ** < 0,01; *** < 0,001.

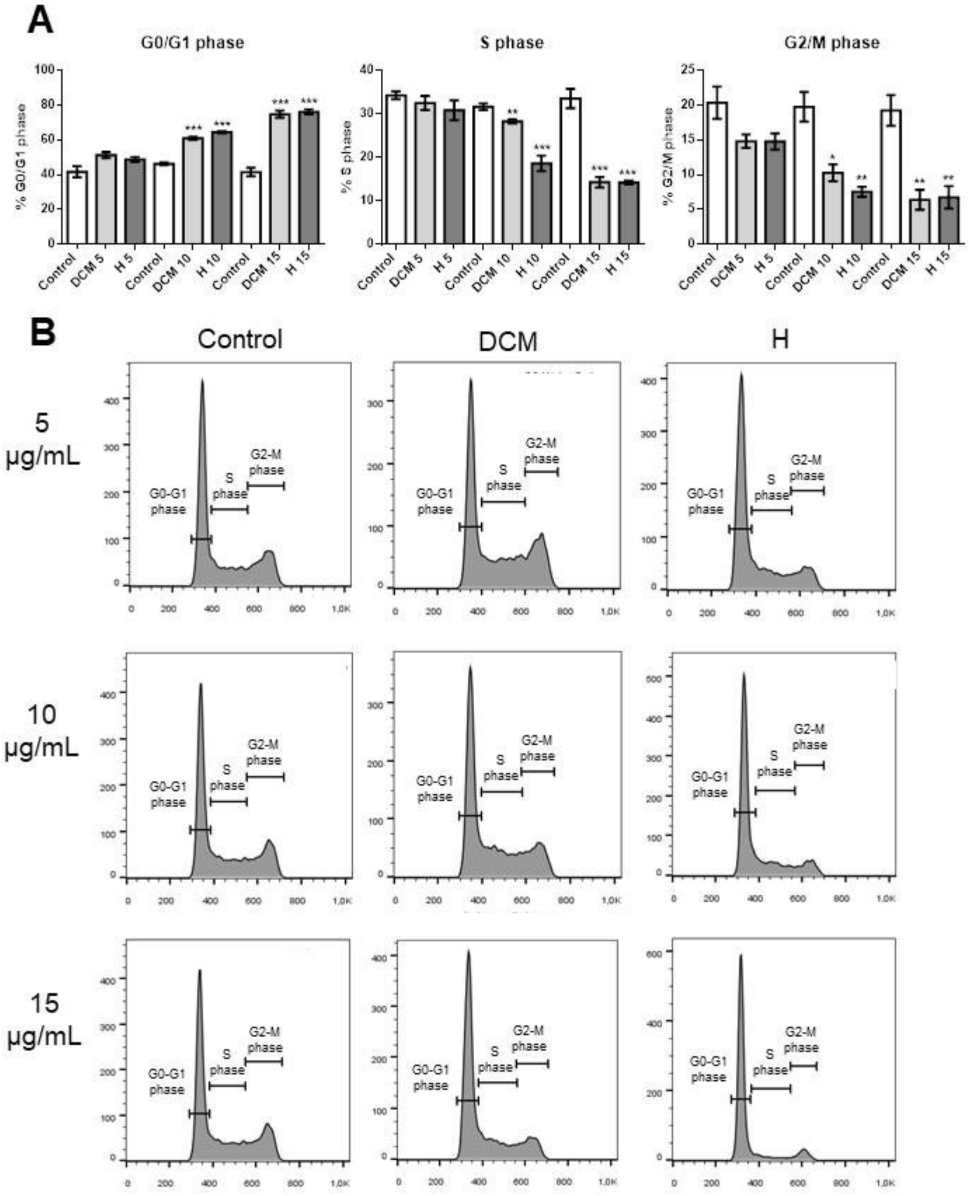

Figure S4. Effects of DCM or H extracts of *L. crithmoides* on OCI-AML3 cell cycle progression. **A.** Bars represent the percentage of cells in G0/G1 (left panel), S (middle panel), or G2/M (right panel) phases after 24 h of treatment with control vehicle (Control) or 5 (DCM 5; H 5), 10 (DCM 10; H 10) or 15 (DCM 15; H 15) µg/mL of the DCM or H extracts, respectively. **B**. Flow cytometry analyses of a representative experiment. Data from three independent experiments are reported as mean ± SEM. * < 0,05, ** < 0,01; *** < 0,001.

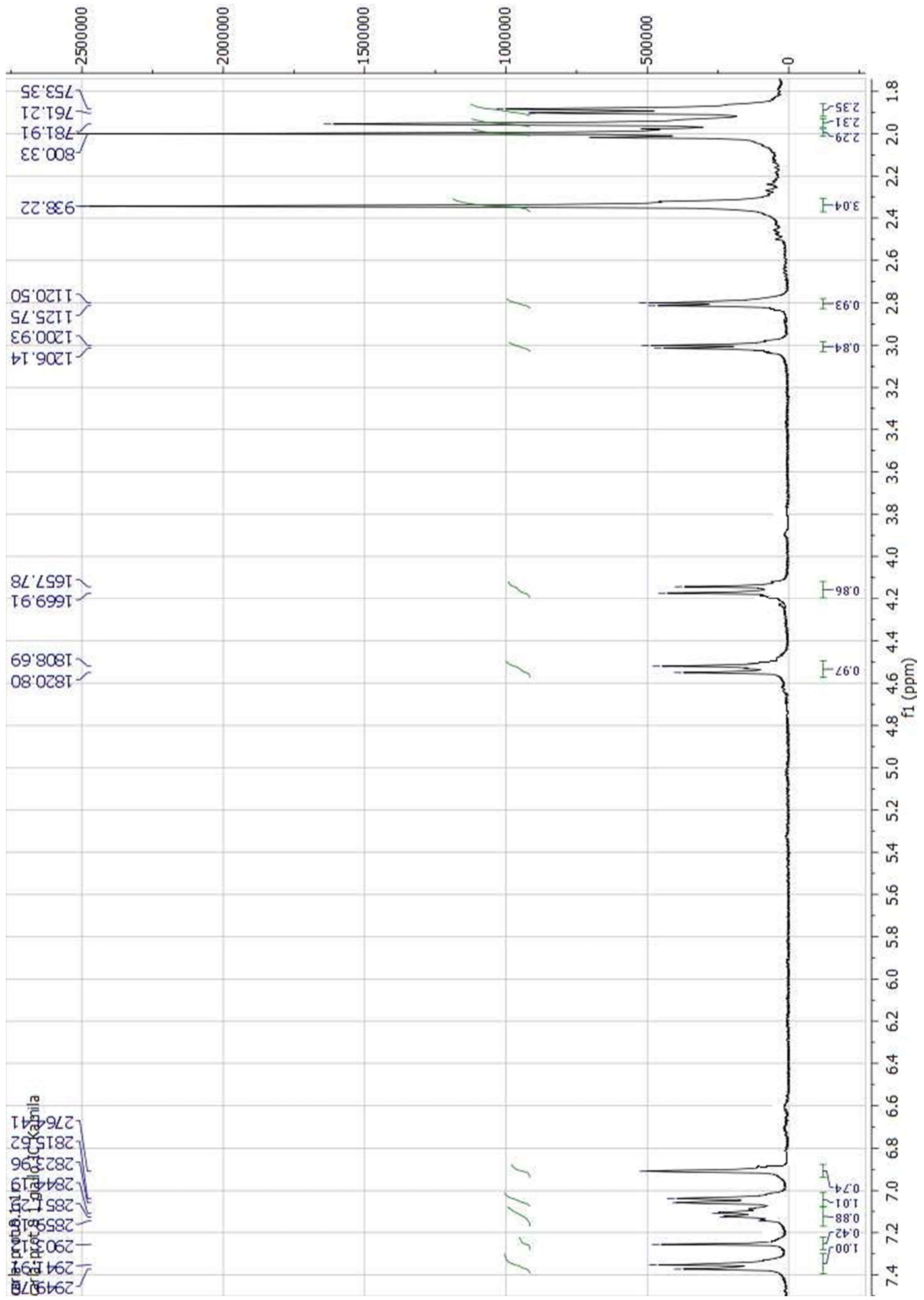

Figure S5. ^{1}H NMR Spectrum of compound **1.**

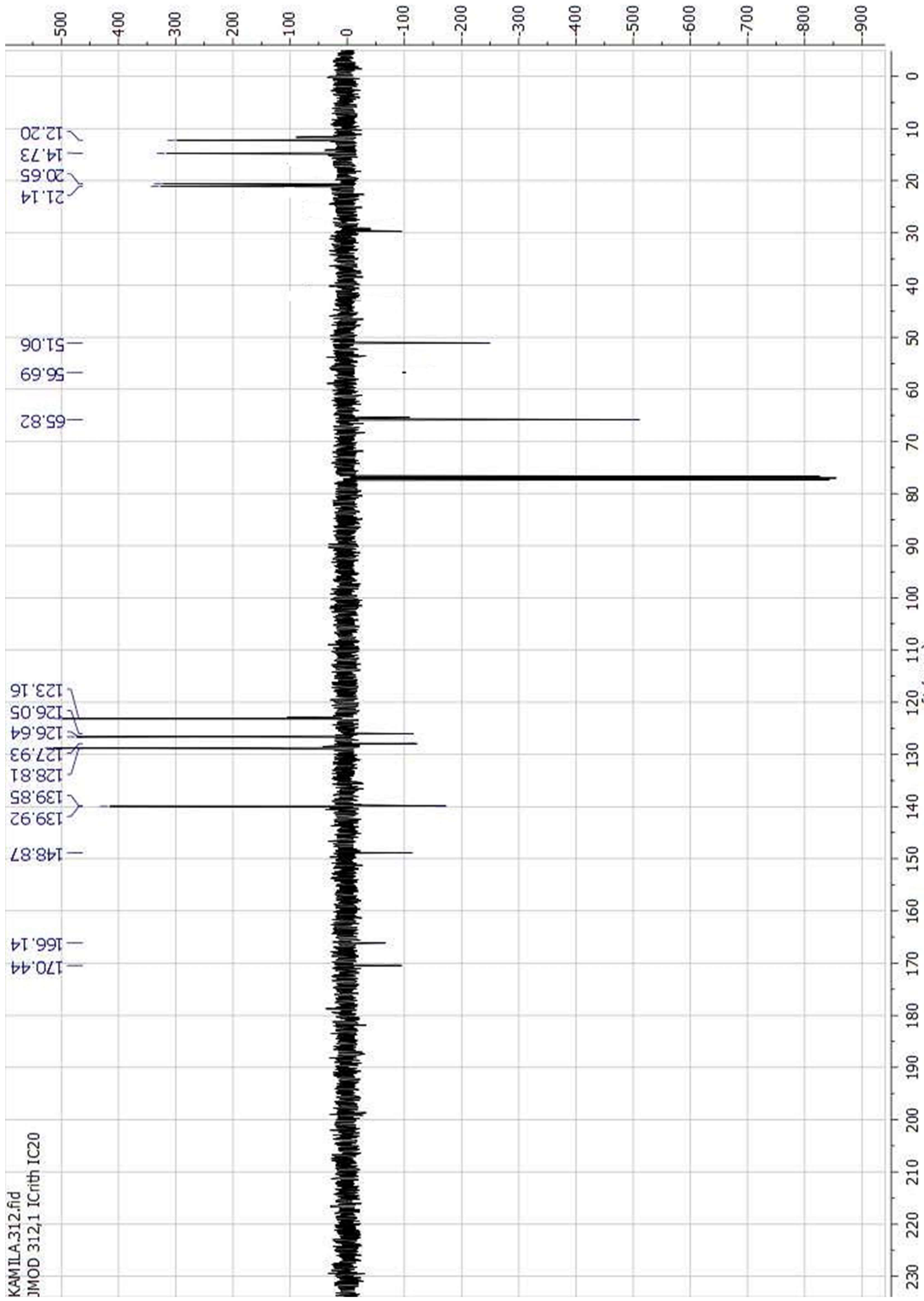

Figure S6. JMODXH spectrum of compound **1**.

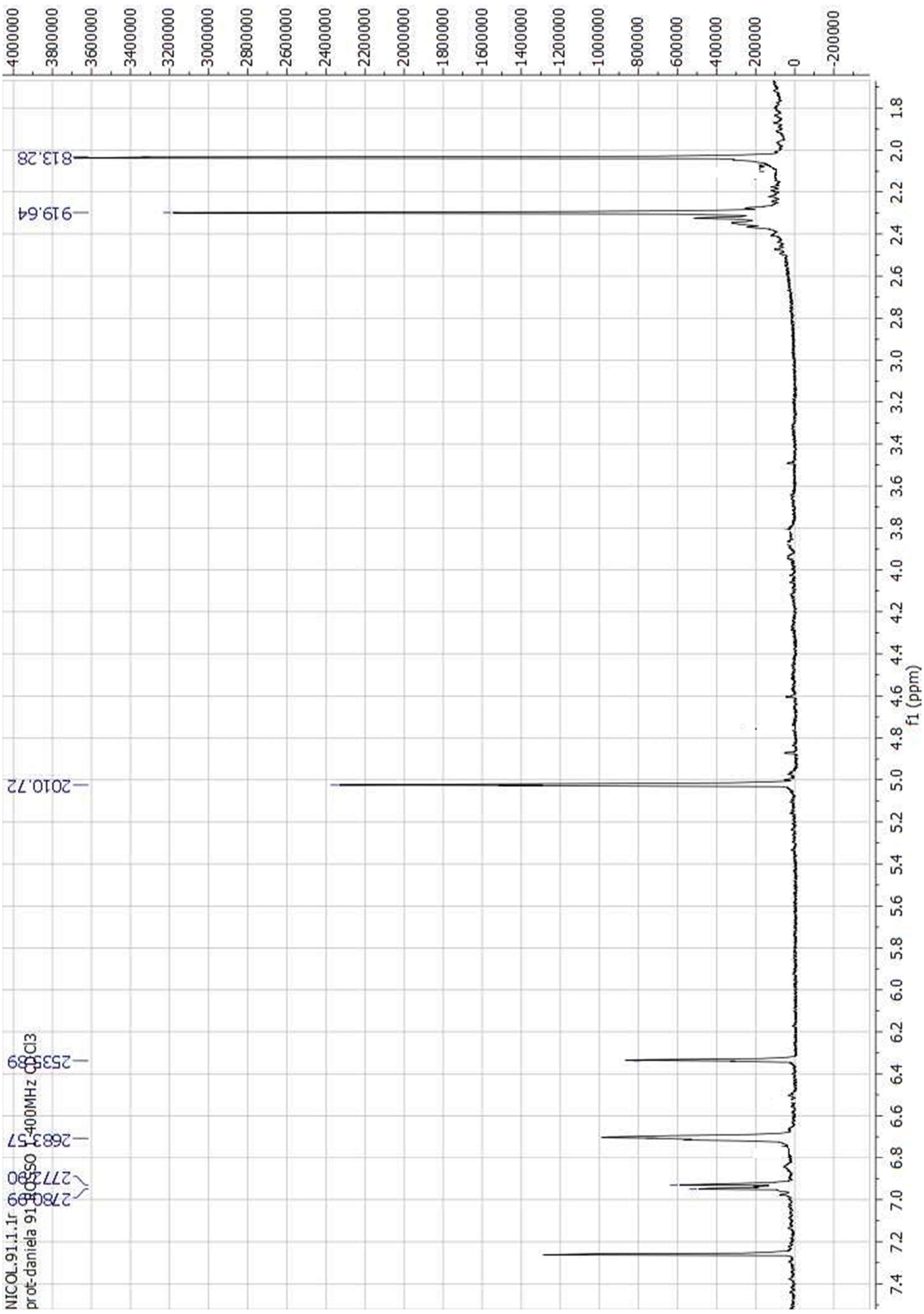

Figure S7. ^{1}H NMR spectrum of compound **2**.

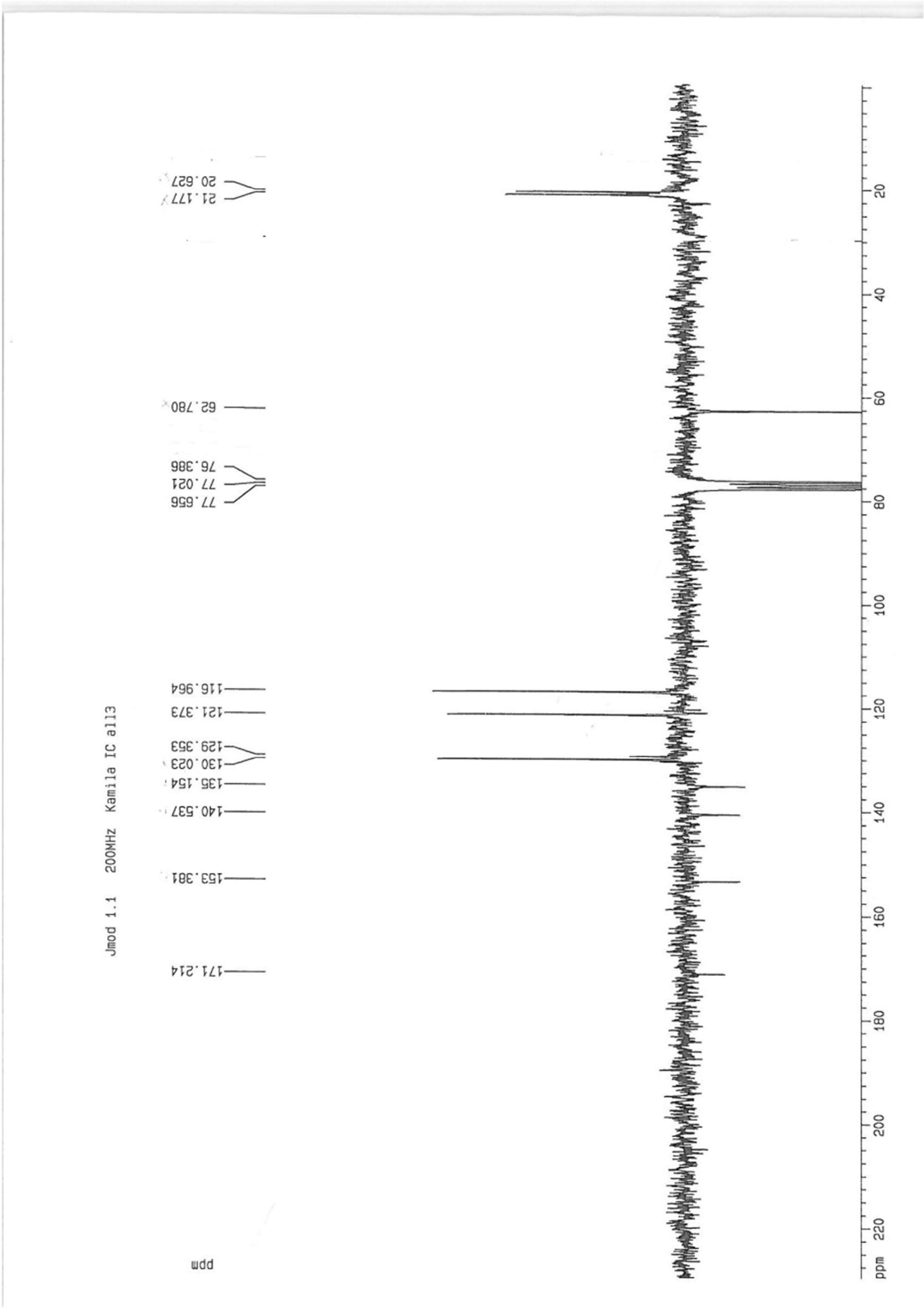

Figure S8. JMODXH spectrum of compound **2**.

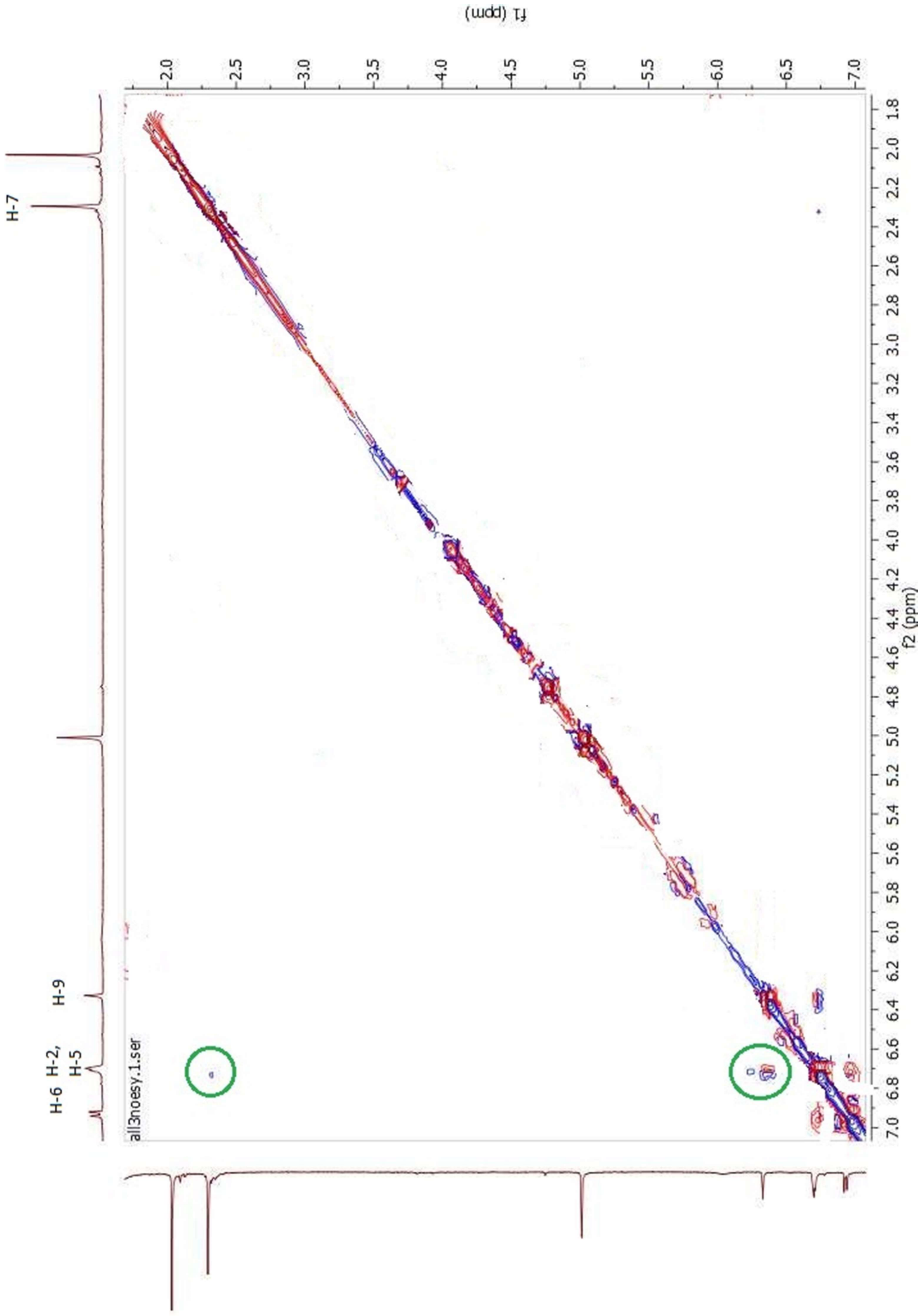

Figure S9. NOESY spectrum of compound **2**.

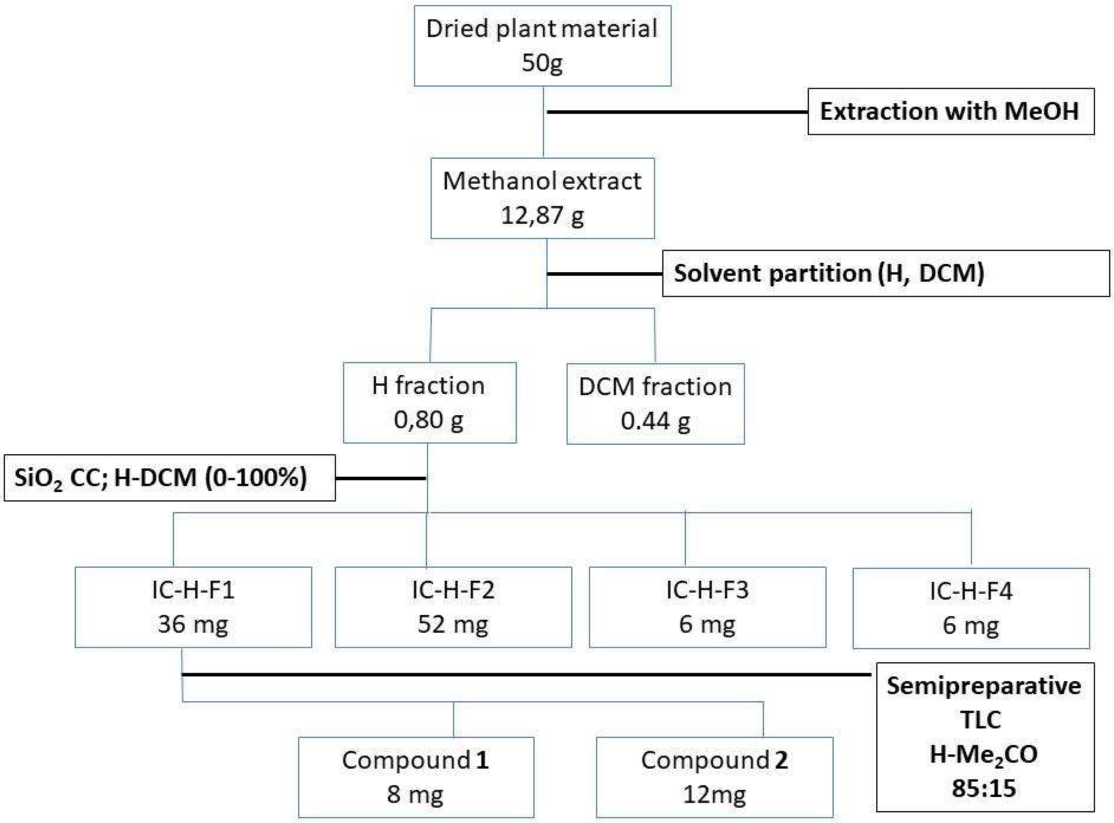

Figure S10. Flow chart of the isolation.
